# Antimalarial Activity of Stem Bark of* Periploca linearifolia* during Early and Established* Plasmodium* Infection in Mice

**DOI:** 10.1155/2018/4169397

**Published:** 2018-01-29

**Authors:** Wubetu Yihunie Belay, Abyot Endale Gurmu, Zewdu Birhanu Wubneh

**Affiliations:** ^1^Department of Pharmacy, Debre Markos Referral Hospital, Debre Markos, Ethiopia; ^2^Department of Pharmacognosy, School of Pharmacy, University of Gondar, Gondar, Ethiopia; ^3^Department of Pharmacology, School of Pharmacy, University of Gondar, Gondar, Ethiopia

## Abstract

**Background:**

In Ethiopia, stem bark of* Periploca linearifolia* is used for the treatment of malaria by the local community and demonstrated antimalarial activity* in vitro*. Despite its* in vitro* antimalarial activity, no scientific study has been carried out to verify its activity* in vivo*. Therefore, the aim of the study was to evaluate the antimalarial activity of* Periploca linearifolia* stem bark extract in mice.

**Methods:**

The dried stem bark of* Periploca linearifolia *was extracted with 80% methanol and evaluated for its antimalarial activity on both early and established* Plasmodium berghei *infected mice. The extract was prepared at graded doses of 200, 400, and 600 mg/kg. Chloroquine and distilled water were administered to the positive and negative control groups, respectively.

**Results:**

The crude extract, at all tested doses, suppressed parasitemia significantly (*p* < 0.05) for 200 and 400 mg/kg and (*p* < 0.001) for 600 mg/kg. The suppression values at these doses were 56.98, 43.33, and 38.17 percent, respectively.* Periploca linearifolia *extract also demonstrated schizonticidal activity in the established malaria infection.

**Conclusion:**

The plant* Periploca linearifolia* has a promising antimalarial activity in mice, supporting its* in vitro *finding. Thus, it could be considered as a potential source to develop new antimalarial agent.

## 1. Background

Malaria, among the most frequent infectious diseases worldwide, causes millions of deaths each year globally. The infection mostly affects children below the age of five. Furthermore, about 99% of the deaths are caused by* P. falciparum* [1]. Growth and multiplication of the* Plasmodium* parasite involve both the vector female* Anopheles* mosquitoes and humans [2]. Malaria in humans is attributable to 5 species of the parasite in the genus* Plasmodium*. These are* P. falciparum, P. vivax, P. malariae, P. ovale*, and* P. knowlesi *[3, 4].

Currently, malaria control is threatened not only by the development of mosquitoes resistant to the available insecticides but also by the currently existing antimalarial agents [1]. Developing antimalarial vaccine is very complex due to antigenic nature of the parasite, its ability to present a different subset of molecules for the immune system, the parasites ability to confuse, hide, and misdirect our immune system, and the possibility of having several plasmodial infections of not only different species but also different strains at the same time [5]. With remarkable efforts, a new antimalarial vaccine candidate RTS,S/AS01 has completed its phase III clinical trial successfully and will be available in Ghana, Malawi, and Kenya starting from 2018 [6]. Traditional medicine through use of natural products plays a great role in the discovery of antimalarial lead compounds and drug candidates like quinine from cinchona bark [7] and artemisinins from* Artemisia annua* [8]. Extract from stem bark of* P. linearifolia* (Asclepiadaceae) demonstrated a promising antiplasmodial activity* in vitro* [9]. Due to the emergence of antimalarial drug resistance and mosquito's resistance to insecticides, there is a high interest in developing new antimalarial drugs [1, 10].

## 2. Methods

### 2.1. Collection and Preparation of the Plant Material

Stem bark of* P. linearifolia* plant was collected from Lay Armachiho Woreda in the vicinity of Tikil Dingay town, North Gondar Zone, 23 km north of Gondar city in December 2016. The plant was botanically identified by a botanist and a voucher specimen was deposited for future reference at the Herbarium of Biology Department, University of Gondar.

### 2.2. Animals

Healthy adult Swiss albino mice of either sex (22–32 g and 6–8 weeks of age) were obtained from the animal house of the School of Pharmacy, College of Medicine and Health Sciences, University of Gondar, Ethiopia. The mice were housed in cages under standard conditions and provided with pellet diet and water ad libitum. The mice were permitted to adapt to the laboratory condition for seven days prior to the beginning of actual experiment. All protocols were performed in accordance with the international animal care and welfare guideline [11]. Indeed, all the experimental procedures were approved by the ethical review board of Department of Pharmacology, School of Pharmacy, University of Gondar.

### 2.3. Parasite Inoculation

Chloroquine-sensitive* P. berghei* (ANKA strain) was obtained from Ethiopian Public Health Institute, Addis Ababa. The parasite was sustained via sequential passage of blood from infected mouse to noninfected ones on a weekly basis.

### 2.4. Drugs and Reagents

Normal isotonic saline (Euro-Med Laboratories, Philippines), absolute methanol (ReAgent Chemical Services, UK), 2% tween 80, hydrochloric acid (Blulux Laboratories, India), citrate dextrose (Deluxe Scientific Surgico, India), Giemsa (Science Lab, USA), ethanol (Nice Chemicals, India), ferric chloride (Fisher Scientific, Co., USA), sodium hydroxide, sulfuric acid (Supertech, India), and Mayer's reagent (Avishkar Lab Tech Chemicals, India) were used.

### 2.5. Extract Preparation

The stem bark was peeled from the plant, air-dried at room temperature under shade, and reduced to appropriate size. A total of 1.2 kg dried stem bark was extracted by maceration (100 g of dried stem bark in 600 ml of 80% methanol) for 72 h. The mixture was filtered using gauze followed by Whatman filter paper number 1 (Whatman®, England). The residue was remacerated for another 72 hour twice and filtered. The combined filtrates were then put under oven at 40°C. After evaporating methanol and some part of the aqueous part, it was transferred to a desiccator for further drying. Finally, the dried extract was kept in a vial and stored in a refrigerator at −4°C until use. 

### 2.6. In Vivo Antimalarial Tests

#### 2.6.1. Parasite Inoculation

*Plasmodium berghei*-infected (20–30% parasitemia) mouse was employed as a donor. The donor mouse was subsequently sacrificed by decapitation and blood collected (via cardiac puncture) into capillary tube containing 0.5% trisodium citrate. Based on parasitemia level of the donor mouse and the red blood cell (RBC) count of normal mouse, the collected blood was diluted with normal saline (0.9%) [12], so that 1 ml blood contains 5 × 10^7^ infected RBCs. Each mouse was injected with 0.2 ml of blood containing 1 × 10^7^* P. berghei*-infected RBCs via the intraperitoneal route.

#### 2.6.2. Grouping and Dosing of Animals

Infected mice were arbitrarily divided into five groups of 5 mice per group. Groups I, II, and III were treated with* P. linearifolia* crude extract (200, 400, and 600 mg/kg), respectively. Groups IV and V were used as control each for chloroquine (25 mg/kg) as positive control and distilled water (2 ml/100 g) as negative control, respectively. Doses were selected based on oral acute toxicity studies.

#### 2.6.3. The Four-Day Suppressive Test

In screening of* P. linearifolia* stem bark extract on an early plasmodium infection, the standard 4-day suppressive test was employed. This test is the most extensively used preliminary test, in which the effectiveness of the extract is evaluated via comparison of blood parasitemia and mouse survival time in extract-treated and untreated mice [13–15]. Treatment was started 3 hours after infection on day 0 and continued daily for four successive days (i.e., from day 0 to day 3). On the fifth day (D4), thin smears of blood were prepared from the tail of each mouse, placed on microscopic slides, fixed in methanol, and Giemsa-stained (10%) at pH 7.2 for fifteen min. Parasitemia was determined via counting the number of parasitized RBCs in four random microscopic fields. Finally, average percent parasitemia and percent suppression were calculated by using the following formula [13, 14]:

(1) % parasitemia=number of parasitized RBCtotal number of RBC×100,% suppression=mean parasitemia of negative control−mean parasitemia of extract treated groupmean parasitemia of negative control×100.

#### 2.6.4. Evaluation on Established Infection (Rane's Test)

The curative effect of the extract was evaluated in accordance with the method described by Ryley and Peters [16]. Standard inocula (1 × 10^7^) of infected RBCs were injected into mice intraperitoneally on day 0. Seventy-two hours later, mice were randomly distributed into groups and dosed once daily for 5 consecutive days. Giemsa-stained thin blood film was prepared from the tail of each mouse daily for 5 days to monitor parasitemia.

#### 2.6.5. Mean Survival Time (MST)

MST was determined arithmetically by calculating the average survival time (in days) of mice starting from date of infection with* P. berghei* for a period of thirty days (D0 to D29).

(2) MST=sum of survival time of all mice in group daystotal number of mice in that group.

#### 2.6.6. Measurement of Packed Cell Volume (PCV)

PCV was considered to predict efficacy of the extract against hemolysis due to rising parasite level associated with malaria. Heparin-coated capillary tubes were employed for blood collection from the tail of each mouse. Blood-filled tubes (up to (3/4)th of the volume) were sealed with sealing clay and then placed in a microhematocrit centrifuge, with the sealed end outwards followed by centrifugation for five minutes at 11,000 rpm. Finally, tubes were removed from the centrifuge and PCV was evaluated via a standard microhematocrit reader. PCV, a measure of the proportion of RBCs to plasma, was measured prior to inoculation with the parasite and at day four.

(3) PCV =volume of erythrocytes in a given volume of bloodtotal blood volume×100%.

#### 2.6.7. Monitoring of Body Weight and Temperature Changes

To examine whether the extract prevented weight loss or not, body weight of each mouse was determined in both the suppressive and curative tests. Weights were recorded on day zero (D0) and day five (D4). Rectal temperature was measured by a digital thermometer ahead of infection 4 hours following infection and then on a daily basis. For the curative test, body weight and temperature of the study mice were measured before infection and after infection (from day 3 to day 7).

### 2.7. Statistical Analysis

The raw data acquired from the experiment was expressed as mean ± SEM and analyzed using SPSS version 20. Statistical significance was determined via one-way analysis of variance (ANOVA) followed by post hoc Tukey's multiple comparison test. Result was considered significant at *p* value < 0.05.

## 3. Results

### 3.1. Preliminary Phytochemical Assay

Phytochemical screening of the hydroalcoholic crude stem bark extract of* P. linearifolia* revealed the presence of alkaloids, saponins, phenolic compounds, tannins, and terpenoids. On the contrary, flavonoids were absent in the extract (Table 1).

### 3.2. Acute Toxicity Study

As shown from the acute oral toxicity study, the extract caused no death of mice at the limit dose (2 g/kg single dose) within the first 24 hours as well as for the subsequent 14 days. Physical and behavioral observations of the experimental mice also discovered no visible signs of toxicity like lacrimation, decrease in appetite, tremors, hair erection, diarrhea, and salivation. This may indicate that the oral LD_50_ of* P. linearifolia *crude extract is higher than 2 g/kg.

### 3.3. Antimalarial Activity

#### 3.3.1. Four-Day Suppressive Test

The results of the study showed that* P. linearifolia* crude extract displayed a significant decrease in parasite count compared to that of the vehicle-treated mice. In addition, it is important to note that the extract dose-dependently suppressed the malaria parasite significantly at all tested doses (200 and 400 mg/kg (*p* < 0.05) and 600 mg/kg (*p* < 0.001)). Moreover,* P. linearifolia* extract at the highest dose used (600 mg/kg) considerably (*p* < 0.05) improved survival date of the study's mice compared to that of the vehicle-treated groups. This effect was much lower than the standard drug (chloroquine 25 mg/kg) that markedly (*p* < 0.001) prolonged survival date of mice (the complete data is presented in Table 2).

#### 3.3.2. Effect on Rectal Temperature and Body Weight Change in the Suppressive Test

The extract at the highest dose (600 mg/kg) caused a significant (*p* < 0.05) decrease in rectal temperature of* P. berghei*-infected mice. Likewise, the standard drug (chloroquine 25 mg/kg) showed a considerable (*p* < 0.001) attenuation in reduction of rectal temperature in the study's mice. The standard drug (chloroquine 25 mg/kg) extensively (*p* < 0.001) prevented malaria-associated body weight loss. In contrast, the extract was unsuccessful in preventing reduction in body weight of mice at all tested doses (Table 3).

#### 3.3.3. Effect of Crude Extract on Packed Cell Volume (PCV) in the Suppressive Test

The crude extract at 400 and 600 mg/kg significantly (*p* < 0.05 and *p* < 0.01, resp.) prevented reduction in PCV compared to that of the vehicle-treated mice. However, the effect was lower than chloroquine 25 mg/kg (*p* < 0.001) (Table 4).

#### 3.3.4. Effect on Established Malaria Infection (Rane's Test)

*P. linearifolia* extract demonstrated a substantial (*p* < 0.01) curative action at the highest dose (600 mg/kg). Mean parasite count at this dose was 41.89 ± 2.77. Even though not comparable to the highest dose, the extract at the lower doses (200 and 400 mg/kg) was also capable of decreasing parasite load to 61.25 ± 8.87 and 64.28 ± 2.66, respectively, while mean parasite count in the untreated control group was 78.75 ± 5.60 (Table 5).

Despite the fact that the standard drug (chloroquine 25 mg/kg) significantly (*p* < 0.001) prolonged survival date of mice (compared to distilled water treated ones), surviving date of mice was not substantially affected by the test substance at all tested doses (Table 5).

#### 3.3.5. Effect of Extract on Body Weight and Rectal Temperature in Rane's Test

*P. linearifolia* extract-treated mice at doses of 400 and 600 mg/kg showed a considerable (*p* < 0.05) reduction in rectal temperature compared to vehicle-treated mice. This effect was much higher (*p* < 0.001) in mice treated with the standard drug. Even though chloroquine at 25 mg/kg significantly (*p* < 0.01) prevented weight reduction, the test extract, at all tested doses, was unable to protect mice against weight loss (Table 6).

#### 3.3.6. Effect of Crude Extract on Packed Cell Volume in Rane's Test

Only 600 mg/kg of the crude extract had significantly prevented PCV reduction as compared to vehicle-treated mice (*p* < 0.05) (Table 7).

## 4. Discussion

The* in vivo* model was preferred for this study because it takes into account the possible prodrug effect and possible contribution of the immune system in eradication of the infection [12].* P. berghei* provides an entrenched experimental model of malaria infection [17] causing pathological symptoms that closely mimic symptoms produced by human malaria [18].

Even though the rodent malaria is not exactly similar to that of the human* Plasmodium *parasites, it is the first step to screen most* in vivo* antimalarial activities of test compounds [13, 14]. Moreover, several of the currently available antimalarial medications (chloroquine, halofantrine, mefloquine, and artemisinin derivatives) have been identified by employing this model [19].

The 4-day suppressive test, which chiefly evaluates the activity of potential antimalarial agents on early malaria infection, and Rane's test, which estimates the curative ability of the test compound on established infection, are the two universally accepted methods in screening of compounds with potential antimalarial activity. In both methods, determination of percent inhibition of parasitemia is the most reliable parameter [13, 14, 16].

It is important to note that percentage parasitemia calculated (from the 4-day suppressive test) revealed the antiplasmodial effect of the extract as shown by the ability of the extract in reducing parasitemia of* P. berghei*-infected mice in a dose-dependent fashion. This may indicate that the plant contains active compounds against the malaria parasite. This antiplasmodial activity exerted by the extract could be attributed to alkaloids and terpenoids contained in* P. linearifolia* crude extract. Alkaloids, as exemplified by quinine, are well known for their antimalarial activity [20]. Methanolic and chloroform extract of* P. linearifolia* was reported to possess antiplasmodial activity* in vitro* [9]. This substantiates our* in vivo* finding. Moreover, a number of chemical compounds have been isolated and identified from* Periploca *genus, such as alpha- and beta-amyrin, lupeol, ß-sitosterol [21], lupene-type triterpenes, and elemane-type sesquiterpenes [21, 22]. In addition, lupeol, ß-amyrin, and ß-sitosterol (terpenoids) are well known for their antimalarial activity against both chloroquine-sensitive and resistant strains of* P. falciparum in vitro* [23]. Therefore, the antiplasmodial activity of* P. linearifolia* in the present* in vivo* study is in agreement with other plants belonging to the same genus. Survival time, in particular at the highest dose, was very much prolonged by the test extract and was associated with a decline in escalated parasitemia level, suggesting its antiplasmodial activity.

Anemia, reduction in body weight, and a decrease in body temperature are typical features of malaria in mice [24]. PCV was calculated to estimate the efficacy of the extract in averting hemolysis due to rising parasitemia level. Basically, the anemia in both human beings and mouse is attributed to the clearance or damage of infected RBCs, coupled with clearance of uninfected RBCs, and inhibition of erythropoiesis by the malaria parasite [25]. This necessitates the analysis of packed cell volume that evaluates the efficacy of the test extract in preventing hemolysis.

The extract, in both the 4-day suppressive test (at 400 and 600 mg/kg) and Rane's test (at 600 mg/kg), substantially prevented reduction in PCV. This reversal of PCV reduction by the test extract may be associated with its ability in reducing parasitemia of infected mice. However, the crude extract was unable to protect mice against weight loss associated with the malaria parasite. This may be due to disturbed metabolic function and hypoglycemia related to malaria infection [26].

Rectal temperature measurements demonstrated that mice were hypothermic particularly in the late infection (Rane's test) with* P. berghei* parasite. Fever is one of the symptoms of malarial infection in humans. On the contrary,* P. berghei*-infected mice were associated with hypothermia rather than pyrexia. In the curative test, the infected mice developed profound hypothermia with rectal temperature falling by as much as 5°C. This extended hypothermia in mice could be associated with the devastating consequence of the malaria parasite on the host which may result in body heat loss and ultimately death of mice [27]. Malaria parasite also affects host carbohydrate, lipid, and protein metabolism [28, 29]. Moreover, a decrease in metabolic rate of* P. berghei*-infected mice accompanied by a corresponding decrease in internal body temperature has been indicated [30]. Therefore, effective antimalarial candidates are likely to prevent the decrease in rectal temperature. As a result,* P. linearifolia* crude extract significantly attenuated the reduction in rectal temperature of mice in contrast to the control (vehicle-treated) group.

The extract at the two lower doses (200 mg/kg and 400 mg/kg) showed variable effect against parasitemia level reduction, that is, the lowest dose (200 mg/kg), though not statistically significant, and showed parasitemia suppression value higher than mice receiving the 400 mg/kg of the extract except on day 5. On the contrary, mice receiving 400 mg/kg of the extract lived longer compared to mice treated with 200 mg/kg of the test extract. This may be due to lower metabolic rate of mice at doses of 200 mg/kg than at 400 mg/kg of the extract (Table 6).

As indicated in the results section of Rane's test, the standard drug (chloroquine 25 mg/kg) started parasitemia reduction following the initial dose. However, this effect was demonstrated by the test extract (at 600 mg/kg) subsequent to the next dose. By and large, results from Rane's test indicated that the test extract (at 600 mg/kg dose) might be useful in curing malaria infection. The effectiveness of the extract both during early infection (suppressive activity) and in the established plasmodium infection (Rane's test) may indicate that* P. linearifolia* could be used as a potential source in the discovery of antimalarial agent from plants. Even though the active antimalarial compound is not elucidated so far, the antimalarial activity of* P. linearifolia* could be due to secondary metabolites (alkaloids, terpenoids, and phenolic compounds) present in the plant. These compounds may produce their antiplasmodial effect either singly or in synergy. Alkaloids, terpenoids, and phenolic compounds are known for their antimalarial activity [31–33].

Potential antimalarial agents with more than 30% suppressive effect on parasitemia which can prolong the survival date of treated mice compared to the control group are often considered effective in standard screening tests [34, 35]. Therefore, the present* in vivo* antimalarial evaluation, in both the suppressive and curative tests, denotes that* P*.* linearifolia* is effective in treating malaria.

## 5. Conclusion 

From the results of this study, it can be concluded that* P. linearifolia* stem bark extract not only is safe to mice but also has a promising antiplasmodial activity. Moreover, the results in this study illustrate that correlations exist between the traditional claim as antimalarial agent and the earlier* in vitro* report.

## Figures and Tables

**Table 1:** Phytochemical screening results of stem bark of *P. linearifolia*.

Phytochemicals	Results
Alkaloids	+
Saponins	+
Terpenoids	+
Flavonoids	−
Phenols	+
Tannins	+

+: present, −: absent.

**Table 2:** Effects of *P. linearifolia* stem bark extract on early infection.

Treatment group	Dose	Parasitemia	% suppression	Survival time
*P. linearifolia *extract	200 mg/kg	24.57 ± 3.29^a1,b3^	38.17 ± 8.28	8.60 ± 0.67
	400 mg/kg	22.52 ± 3.40^a1,b2^	43.33 ± 8.56	11.60 ± 2.56
	600 mg/kg	17.10 ± 2.88^a3,b1^	56.98 ± 7.25	16.4 ± 4.20^a1,b2^
Distilled water	10 ml/kg	39.74 ± 4.99	-	6.80 ± 0.37
Chloroquine	25 mg/kg	0.00 ± 0.00^a3^	100.00 ± 0.00	30.00 ± 0.00^a3^

Data are expressed as mean ± SEM; *n* = 5;  ^a^compared to vehicle (distilled water),  ^b^compared to chloroquine (25 mg/kg), ^1^*p* < 0.05, ^2^*p* < 0.01, and ^3^*p* < 0.001.

**Table 3:** Rectal temperature and body weight change of infected mice treated with *P. linearifolia* extract in the suppressive test.

Extract	Dose	Temperature (°C)			Weight		
		D0	D4	% Change	D0	D4	% change
Extract (mg/kg)	200	37.18 ± 0.32	36.46 ± 0.2	−1.93	28.8 ± 1.11	26.74 ± 0.92	−7.15
	400	37.68 ± 0.05	37.04 ± 0.16	−1.69	27.72 ± 1.00	25.76 ± 1.20	−7.07
	600	37.58 ± 0.09	37.20 ± 0.11	−1.01^a1^	26.9 ± 1.23	24.92 ± 1.38	−7.36
Chloroquine	25	36.76 ± 0.33	36.9 ± 0.24	0.38^a3^	27.52 ± 1.70	27.38 ± 1.42	−0.50^a2^
Distilled water	10 ml	36.76 ± 0.22	35.16 ± 0.29	−4.35	30.42 ± 0.72	26.56 ± 0.89	−12.68

Data are expressed as mean ± SEM; *n* = 5;  ^a^compared to distilled water treated group, ^1^*p* < 0.05, ^2^*p* < 0.01, and ^3^*p* < 0.001. D0: value before treatment on day 0; D4: value after treatment on day four.

**Table 4:** PCV of *P. linearifolia*-treated mice during the 4-day suppressive test.

Extract	PCV0	PCV4	% change
D/water 10 ml/kg	53 ± 0.44	47.4 ± 0.59	−10.56 ± 0.59
Chloroquine 25 mg/kg	50.8 ± 0.65	48.8 ± 0.25	−3.93 ± 0.25^a3^
PL200 mg/kg	51 ± 0.66	46.8 ± 0.32	−8.23 ± 0.32^b2^
PL400 mg/kg	52 ± 0.46	48.4 ± 0.21	−6.92 ± 0.21^a1^
PL600 mg/kg	52.4 ± 0.83	49.4 ± 0.30	−5.72 ± 0.30^a2^

Data are expressed as mean ± SEM; *n* = 5;  ^a^compared to distilled water treated group, ^b^compared to chloroquine 25 mg/kg, ^1^*p* < 0.05, ^2^*p* < 0.01, and ^3^*p* < 0.001. PCV0: pretreatment value on day 0; PCV4: posttreatment value on day four; PL: crude extract of *P. linearifolia*; PCV: packed cell volume.

**Table 5:** Parasitaemia and survival date of infected mice treated with *P. linearifolia* extract in Rane's test.

Dose	Parasitemia level					% inhibition	Survival time
	Day 3	Day 4	Day 5	Day 6	Day 7		
PL200	10.08 ± 2.06	28.74 ± 5.56	49.36 ± 5.90	53.99 ± 5.74	61.25 ± 8.87^b2^	22.22 ± 11.26	8.40 ± 0.50^b2^
PL400	11.01 ± 1.80	34.01 ± 3.90	47.60 ± 2.01	56.47 ± 3.52	64.28 ± 2.66^b2^	18.37 ± 3.38	8.80 ± 0.66^b2^
PL600	8.64 ± 1.41	25.02 ± 4.43	36.01 ± 4.22	38.75 ± 3.58	41.89 ± 2.77^a2,b2,c1^	46.79 ± 3.52	10.40 ± 1.36^b2^
CON.	11.52 ± 1.86	37.39 ± 5.74	61.62 ± 6.87	66.96 ± 5.70	78.75 ± 5.60	-	7.60 ± 0.24
CQ25	9.56 ± 3.04	9.41 ± 3.71	1.10 ± 0.40	0.32 ± 0.23	0.00 ± 0.00^a2^	100.00 ± 0.00	30.00 ± 0.00^a2^

Data are expressed as mean ± SEM; *n* = 5;  ^a^compared to negative control (distilled water),  ^b^to CQ, and  ^c^to PL400. ^1^*p* < 0.05 and ^2^*p* < 0.001. CQ: chloroquine; CON: negative control; PL: crude extract of *P. linearifolia*. Numbers refer to doses used in mg/kg.

**Table 6:** Body weight and rectal temperature changes of infected mice treated with* P. linearifolia* extract in Rane's test.

Dose	Weight			Temperature		
	W0	W4	% change	T0	T4	% change
PL200	27.90 ± 1.08	24.74 ± 0.80	−11.32^b2^	36.98 ± 0.17	34 ± 0.46	−7.67^b2^
PL400	27.08 ± 1.00	23.68 ± 0.89	−12.55^b2^	36.64 ± 0.12	34.32 ± 0.35	−6.33^a1,b1^
PL600	26.92 ± 1.29	24.62 ± 0.79	−8.54^b1^	37.12 ± 0.11	34.52 ± 0.43	−7.00^a1,b2^
CON.	27.26 ± 1.13	23.64 ± 1.27	−13.27	36.74 ± 0.21	32.44 ± 0.20	−11.70
CQ25	26.08 ± 1.20	26.56 ± 1.39	1.9^a2^	36.64 ± 0.17	36.22 ± 0.24	−1.14^a3^

Data are expressed as mean ± SEM; *n* = 5;  ^a^compared to control;  ^b^compared to CQ; ^1^*p* < 0.05, ^2^*p* < 0.01, and ^3^*p* < 0.001. CQ: chloroquine; CON: control; PL: crude extract of *P. linearifolia*. Numbers refer to dose in mg/kg.

**Table 7:** PCV of infected mice treated with crude extract of *P. linearifolia* in Rane's test.

Extract	PCV0	PCV4	% change
D/water 10 ml/kg	49.8 ± 0.29	37.2 ± 1.07	25.3 ± 2.0
Chloroquine 25 mg/kg	47.4 ± 0.81	44.4 ± 0.67	−6.32 ± 0.70^a3^
PL 200 mg/kg	48.8 ± 0.51	40.2 ± 0.43	17.62 ± 0.59^b1^
PL 400 mg/kg	48.4 ± 0.60	39.0 ± 0.40	−19.42 ± 0.48^b2^
PL 600 mg/kg	51.0 ± 0.74	44.4 ± 0.80	−12.94 ± 0.61^a1^

Data are expressed as mean ± SEM; *n* = 5;  ^a^compared to d/water;  ^b^compared to CQ; ^1^*p* < 0.05, ^2^*p* < 0.01, and ^3^*p* < 0.001. PL: *P. linearifolia* crude extract; PCV: packed cell volume.

## Additional Points

*Recommendation*. Further isolation and characterization of the active compound(s) responsible for its antimalarial activity are required. Subacute and chronic toxicity studies need to be further conducted.
